# Complete genome sequence of the *Microbacterium enclense* bacteriophage phiMiGM15

**DOI:** 10.1128/mra.00302-24

**Published:** 2024-05-03

**Authors:** Erika Camacho-Beltrán, Juan José Morales-Aguilar, Melina López-Meyer, Gabriel Rincón-Enríquez, Evangelina Esmeralda Quiñones-Aguilar

**Affiliations:** 1 Laboratorio de Fitopatología, Unidad de Biotecnología Vegetal, Centro de Investigación y Asistencia en Tecnología y Diseño del Estado de Jalisco, Zapopan, Jalisco, Mexico; 2 Instituto Politécnico Nacional, Centro Interdisciplinario de Investigación para el Desarrollo Integral Regional (CIIDIR-Unidad Sinaloa), Juan de Dios Bátiz Paredes 250, Guasave, Sinaloa, Mexico; 3 Universidad Autónoma de Occidente, Unidad Regional Guasave. Avenida Universidad S/N Colonia Villa Universidad, Guasave, Sinaloa, Mexico; Queens College, Queens, New York, USA

**Keywords:** *Microbacterium enclense*, diseases of bean crops, biological control

## Abstract

We characterized the complete genome sequence of phiMiGM15, a lytic bacteriophage with siphovirus morphology that infects *Microbacterium enclense*. Its 48.6 kb genome contains 81 putative genes and shows coverage of 28% with 82.26% of nucleotide identity to *Microbacterium* phage Caron accession number OQ190481.1.

## ANNOUNCEMENT

**Fig 1:**
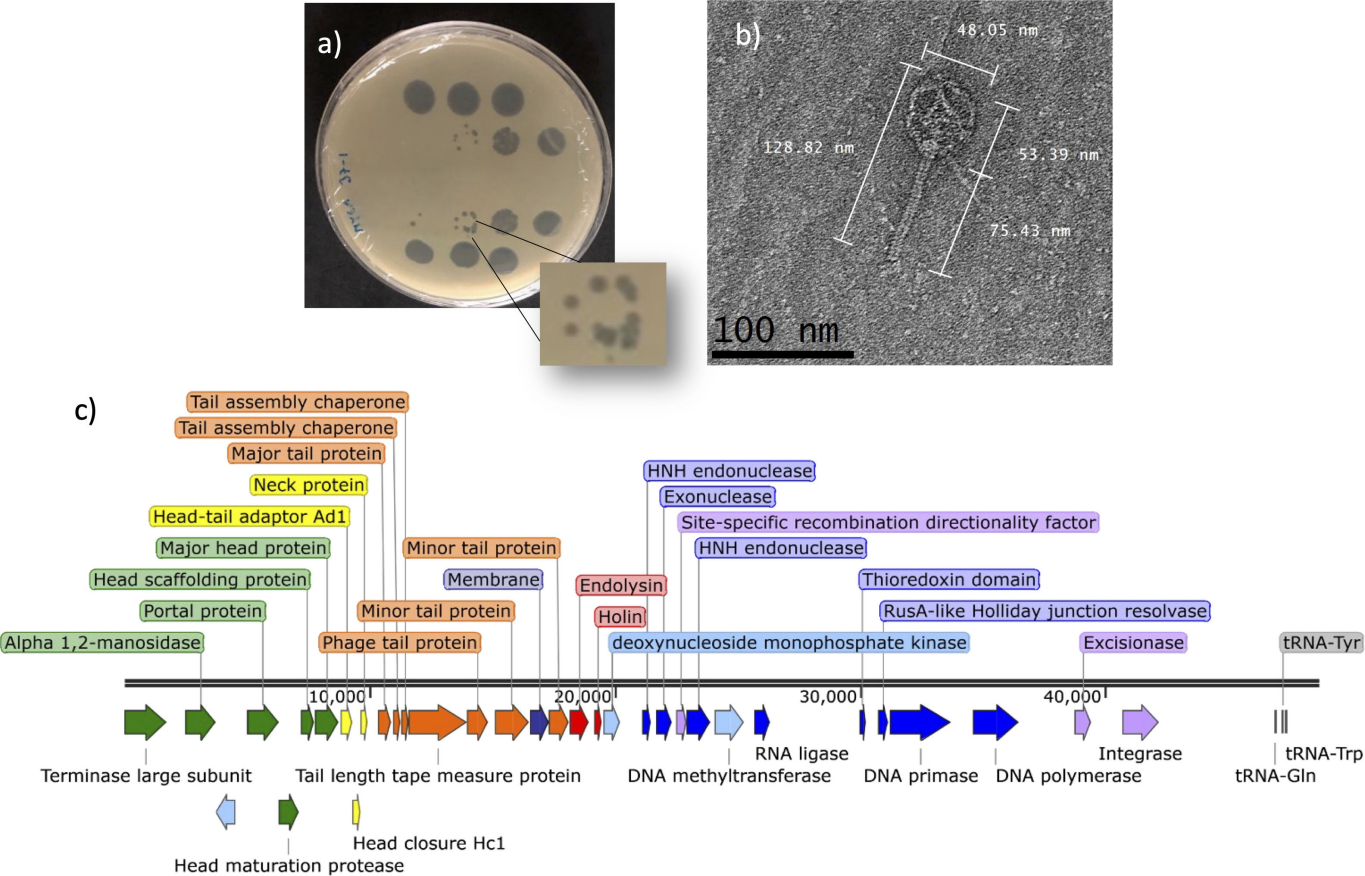
Characterization of the *Microbacterium* bacteriophage phiMiGM15. (a) phiMiGM15 forms small plaques with a clear center surrounded by a turbid border. single plaques (1 mm) were isolated and purified two times. (b) An electron microscopic image highlights the siphovirus morphology of phiMiGM15, with an icosahedral capsid (diameter, ~48.05 nm) attached to a noncontractile tail (length ~75.43 nm). The sample was viewed at an accelerating voltage of 200 kV with a LaB6 transmission electron microscope (JEOL, JEM 2100) after it was fixed with 1% glutaraldehyde on a freshly glow-discharged Formvar/carbon-coated copper grid for 10 min and stained with 1% aqueous uranyl acetate for 1 min. (c) The phiMiGM15 genome shows 35 annotated putative protein-coding genes with known functions. Colors are assigned to expected functions according to annotation information. Green, head and packing; yellow, connector; orange, tail; indigo, membrane; red, lysis; blue, RNA and nucleotide metabolism; violet, integration and excision; light blue, other functions; gray, tRNA. The image was created with SnapGene software.

Bacteriophages are a group of viruses that infect bacteria. Bacteriophages could be a sustainable alternative to copper salts and antibiotics for phytopathogens’ bacterial biological control (1). Bacteriophage phiMiGM15 (family *Autographiviridae*) was isolated from soil samples collected on bean fields with symptoms associated with bacterial wilt disease (Guasave, Sinaloa, México, 25.544444 N, 108.376389 W). The soil sample was suspended in peptone yeast glycerol medium, inoculated with *Microbacterium enclense* (laboratory strain BV37), and incubated (24 h, 28°C). The resulting slurry was centrifuged (8,000 × *g*, 20 min), and the supernatant was filtered (0.22 µm). Phages in the supernatant were isolated in double-layer plaque assays (0.7% agar) (2). phiMiGM15 forms small plaques with a clear center surrounded by a turbid border (Fig. 1a). The phage morphology was visualized using negative staining with uranyl acetate (3). phiMiGM15 exhibits a siphovirus morphology (Fig. 1b). Phage genomic DNA was extracted using the phenol-chloroform method (4). The nature of the genome was characterized using DNase I (04536282001, Roche), RNase A (R6148, Sigma-Aldrich), and S1 nuclease (M5761, Promega) according to the manufacturers’ instructions, presenting double-stranded DNA genome. For the complete genome analysis, a DNA genomic library was prepared with the MiSeq Reagent Nano Kit v2 (MS-103–1001, Illumina). High-throughput sequencing was performed with a Miseq instrument (SY-410-1003, MiSeq; Illumina) with a 2 × 150 bp maximum read length, which resulted in 3,353,744 paired-end 150 bp raw reads. The genome was assembled using the Illumina A5-miseq pipeline v20160825 (5). A contig of 48,645 bp with a G+C content of 69.37%, and average coverage of 4,910 was obtained, then the accuracy and completeness of the genome were checked using QUAST v5.2 (6), and PhageScope v1.1 (7) showing 100% of completeness assessment. The termini were analyzed by PhageTerm (Galaxy v1.0.12) (8), resulting in redundant ends multiple permuted. The genome was manually reviewed, then annotated using pharokka v1.2.0 (9) embedded in Galaxy server v1.3.2 using terminase large subunit re-orientation mode (10) and PhageScope v1.1 (7). Default parameters were used in all software except pharokka v1.2.0. Eighty-one putative genes were identified. Putative functions could be assigned to 35 genes, including head and tail assembly genes, endolysin, and holin genes (involved in host cell lysis) (11), endonuclease, exonuclease genes, RNA and nucleotide metabolism, integration, and excision, and tRNA (Fig. 1c). A BLAST analysis was performed in some of the proteins in phiMiGM15 such as the major head protein, holin, and DNA polymerase presenting 85%, 64.52%, and 81.26% of amino acid identity to *Microbacterium* phage Caron proteins accession numbers WDS52032.1, WDS52048.1, and WDS52076.1, respectively, also the major tail protein show 79% of amino acid identity to *Microbacterium* phage barnstormer protein accession number WDS51648, and the endolysin showing 71.13% of amino acid identity to *Microbacterium* phage Honk protein QWY81842.1. BLAST analysis of the complete genome shows a coverage of 28% with 82.26% of nucleotide identity to *Microbacterium* phage Caron OQ190481.1, suggesting, phiMiGM15 is a completely new *Microbacterium* phage and similar in some proteins to others *Microbacterium* phages. phiMiGM15 can be used in biological control programs for diseases of bean crops.

## Data Availability

The genome sequence of bacteriophage phiMiGM15 was deposited under GenBank accession number PP393683. The raw sequence reads are available in the SRA database with the accession number SRX23801183 (BioProject accession number PRJNA1081932).
